# Increased expression of SRPK1 (serine/arginine-rich protein-specific kinase 1) is associated with progression and unfavorable prognosis in cervical squamous cell carcinoma

**DOI:** 10.1080/21655979.2022.2034705

**Published:** 2022-02-22

**Authors:** Zhanfei Dong, Xuezhi Chang, Li Xie, Yina Wang, Youxiang Hou

**Affiliations:** a Department of Nuclear Medicine, The Affiliated Cancer Hospital of Xinjiang Medical University, Urumqi, Xinjiang, China; b Department of Radiation Oncology, Yili Friendship Hospital, Yili, Xinjiang, China; c Department of Radiation Gynecological Oncology, The Affiliated Cancer Hospital of Xinjiang Medical University, Urumqi, Xinjiang, China

**Keywords:** Cervical squamous cell carcinoma, serine/arginine-rich protein-specific kinase 1, prognosis, proliferation, invasion

## Abstract

Previous studies suggest that SRPK1 (serine/arginine-rich protein-specific kinase 1) is involved in tumorigenesis and closely related to unfavorable outcomes. However, its expression pattern in cervical squamous cell carcinoma (CESC) remains uncovered. In this study, we initially investigated the clinical significance and function of SRPK1 in human CESC. Data mining and analysis on SRPK1 mRNA expression in CESC samples were conducted using TCGA database, which indicated that SRPK1 mRNA was significantly upregulated in CESC samples. Protein expression of SRPK1 was tested by immunohistochemistry in a retrospective cohort (n = 122), revealing a higher SRPK1 protein abundance in CESC specimens whose aberrant up-regulation was obviously related to worse survival. Cox proportional hazards regression analysis further confirmed the role of SRPK1 as an independent prognostic factor of CESC. Cellular experiments validated that SRPK1 may function through enhancing CESC proliferation, migration, and invasion. In conclusion, aberrant up-regulation of SRPK1 is remarkably related to progression and unfavorable prognosis of CESC, which can serve as a novel prognostic biomarker and therapeutic target for CESC.

## Introduction

Cervical cancer accounts for the second most prevalent gynecological cancer worldwide [1–4], leading to approximately 530,000 new cases and 270,000 deaths every year [5,6]. Cervical squamous cell carcinoma (CESC) comprises more than 75% of all cervical cancers. The average 5-year overall survival rate is about 72% in all CESC patients [7]. As most malignancies, the prognosis of CESC is largely dependent on its clinical stage, the FIGO (The International Federation of Gynecology and Obstetrics) stage. For example, the 5-year overall survival of cervical cancer was about 91% for FIGO stage I cases, while decreased to 16% for those with FIGO stage IV [7]. Of note, the median disease recurrence time after surgical resection was approximately 14.8 months for stage I–III cases [8], and the median post-recurrence survival time was only 28.4 months [9]. Therefore, it is critical to identify novel biomarkers to predict disease recurrence, which will be also invaluable for directing individualized therapy.

Splicing factor refers to a protein family which can remove introns from pre-mRNA in spliceosomes. Alternative removal of introns and binding of exons is one of the most important processes to generate numerous mature transcripts from a limited number of human genes, thus contributing to the protein diversity. Involvement of splicing factors in human malignancies has been well-summarized [10–12]. Like most proteins, the function of splicing factors was precisely regulated by post-translational modifications. For example, phosphorylation plays a critical role on modulating the subcellular localization and activity of splicing factors, thus participating in tumorigenesis [13–15]. There are three predominant kinase subfamilies targeting splicing factor in human cells, including serine-arginine protein kinases (SRPK), the CDC-like kinases, as well as the pre-mRNA processing factor 4 kinase [16]. Among them, SRPK1 represents the first reported splicing kinase by Gui et al. in 1994, which was initially regarded to participate in cell cycle [17].

As one of the most important kinases on regulating alternative splicing, the role of SRPK1 in malignancies has been discovered recently [18]. For example, SRPK1 was found to be aberrantly upregulated in colon adenocarcinoma [19], pancreatic cancer [20], prostate cancer [21], and hepatocellular carcinoma [22]. Hyper-activation of SRPK1 can also promote the progression of triple-negative breast cancer [23]. Therefore, SRPK1 was recognized as a potential target for anti-tumor therapies [24]. However, it may exert distinct functions in different tumor types [25].

Till now, there is no study reporting the expression and function of SRPK1 in cervical cancers. Considering its reported role in other malignancies, we hypothesized that SRPK1 may participate in cervical cancer progression. Here, we demonstrated that SRPK1 was upregulated in CESCs on both mRNA and protein levels, and its upregulation was remarkably correlated with tumor progression as well as unfavorable prognosis. By using knockdown and overexpression strategies, we further confirmed that SRPK1 can facilitate proliferation, migration, and invasion of CESC cells (C-33A and SW756 cell lines). Our findings provided the first evidence that SRPK1 can serve as a novel prognostic factor for CESC. In addition, we validated that silencing SRPK1 can significantly attenuated proliferation and invasion of CESC, thereby providing a potential direction for CESC treatment.

## Patients and methods

### Online data mining

The mRNA transcription level of SRPK1 was first retrieved from TCGA (The Cancer Genome Atlas) database [26] using the Gene expression profiling interaction analysis (GEPIA) online server (http://gepia.cancer-pku.cn) [27]. The mRNA levels of SRPK1 in CESC tissues (n = 306) and normal tissues (n = 13) were compared using Student’s t-test. Besides, we compared the survival differences of CESC patients stratified by the transcription levels of SRPK1 mRNA using Kaplan–Meier method.

### Cohort enrollment and ethics

This study was conducted in accordance with the Declaration of Helsinki and approved by the Ethics Committee of Xinjiang Medical University (No. K-2019010). Written informed consents were obtained from all participants. We collected a retrospective cohort containing 122 early stage CESC (FIGO stage I–II) cases from our hospital. All cases underwent curative surgical resection, and their resected tumor specimens were formalin-fixed and paraffin-embedded. The median diagnostic age was 54 years old, ranging 31–69 years old. The median follow-up time was 41 months, ranging 9–109 months. Disease-free survival (DFS) was defined as the period from diagnostic to first evidence of recurrence or death. Overall survival (OS) referred to the period between disease diagnoses to the time of death.

### Immunohistochemistry (IHC) staining

Expression of SRPK1 was assessed by a standard IHC method [28]. Anti‐human SRPK1 monoclonal antibody (1:500, sc-100443, Santa Cruz BioTechnology) was selected as the primary antibody, and anti‐mouse antibody (1:5000, ab6708, Abcam) was used as the secondary antibody. IHC data were evaluated by two independent pathologists who were blinded to patients’ information, a consensus was required to be obtained in case of divergence. The staining was assessed by both staining intensity and proportion of stained cells.

Accordingly, staining intensity was scored as no staining (0 point), weak (1 point), moderate (2 points), or strong (3 points). Proportion of the stained cells was classified as negative (0 point), <33% (1 point), 33%‐66% (2 points), or >66% (3 points). The final IHC staining score was obtained by the product of the two scores. An overall score of 3 or less was defined as low SRPK1 expression, and that of 4 scores or more was defined as high SRPK1expression.

### Cell culture and transfection

Two CESC cell lines, C-33A and SW756, were obtained from ATCC (American Type Culture Collection) and cultured in Dulbecco’s Modified Eagle Medium (Gibco, UA, USA) supplemented with 10% fetal bovine serum (FBS, Gibco) in cell culture incubator with 5% CO2 at 37°C.

Three shRNAs were designed and synthesized by GenePharma (Shanghai, China), including scrambled control shRNA, SRPK1-shRNA#1 (5’-CCGGCCATAACTAAAGGATCAGGATCTCGAGATCCTGATCCTTTAGTTATGGTTTTT-3’), and SRPK1-shRNA#2 (5’-CCGGGTGGCAATGAAAGTAGTTAACTCGAGTTTAACTACTTTCATTGCCACTTTTT-3’). Overexpression plasmid of SRPK1 was synthesized by inserting the coding sequence of SRPK1 into the pcDNA3.1 vector, using the blank vector as control. Transfection was achieved by FuGENE® 6 Transfection Reagent (Promega, USA) according to the manufacturer’s protocol.

### Western blot

Total protein was extracted from transfected cells using radioimmunoprecipitation assay buffer (Beyotime, China) supplemented with protease inhibitor cocktail (Roche, USA). After protein quantification with a bicinchoninic acid kit (Thermo Fisher Scientific, USA), equal amount of protein samples were separated using sodium dodecyl sulfate polyacrylamide gel electrophoresis and transferred onto a polyvinylidene difluoride membrane using the Bio-Rad system (Bio-Rad, USA). After an overnight incubation with an anti-SRPK1 antibody (1:2000, sc-100443, Santa Cruz) or an anti-beta-actin antibody (1:2000, sc-47778, Santa Cruz) at 4°C, membranes were incubated with secondary antibody (1:5000, ab6708, Abcam) at room temperature for an additional 1 h. The protein expression level was finally evaluated by using the Chemiluminescent Substrate Reagent kit (Thermo Fisher Scientific) [29] using Tanon 4200SF Multi-Function Imaging System (Shanghai Tianneng Technology Co., Ltd., China). Finally, images were semi-quantified using the Image J Software (NIH, USA).

### Cell proliferation assay

Cell viability was evaluated using a MTT (3-(4,5-Dimethylthiazol-2-yl) method. Briefly, transfected C-33A or SW756 cells were cultured for 48 hours and inoculated into 96-well plates at 3000 cells/well. After cultured for different time points (8h, 24 h, 48 h, 72 h, and 96 h), the medium was discarded and 100 μl MTT reagent were added to each well and cultured for another 4 h. Then 200 μl dimethylsulfoxide reagent was added to resolve the MTT crystals. Finally, the plates were sent to a microplate reader (Multiskan Fc Photometer, Thermo Fisher Scientific, USA) to measure absorbance at 570 nm wavelength.

### Migration and invasion assays

Migration capacity was evaluated by using the Transwell method. Briefly, 5,000 cells in 200 μl serum-free medium were seeded into the upper chamber, while 600 μl medium containing 10% FBS was supplied to the lower chamber. After cultured at 37°C in incubator for 24 h, cells were fixed with 4% paraformaldehyde and stained with 0.5% crystal violet. The migrated cells were counted under a light microscope (Nikon Eclipse Microscope Ti, Nikon, Japan). The invasion assay was conducted similarly except that the chamber was pre-coated with Matrigel (BD Biosciences, USA) and the seeding cell number was 20,000 cells in 200 μl serum-free medium.

### Statistical analyses

All statistical analyses were performed using IBM® SPSS® Statistics Software version 19.0. Chi-square test was used to evaluate the correlation between SRPK1 expression and the clinicopathological characteristics of CESC patients. Survival curves were plotted using the Kaplan–Meier method and compared by log-rank test. The independent prognostic significance of SRPK1 as well as other variables were assessed using a Cox proportional hazards regression model. All cellular experiments were repeated for three independent times and data were presented as mean + standard deviation (SD). Student’s t-test was used for comparisons between two groups, while Tukey’s test was used with One-way ANOVA for comparisons among multiple groups. P < 0.05 was considered statistically significant.

## Results

We hypothesized that SRPK1 may be aberrantly expressed in CESC and may participate in cervical cancer progression. Here, we first demonstrated that SRPK1 was upregulated in CESCs on both mRNA and protein levels, and its upregulation was remarkably correlated with tumor progression as well as unfavorable prognosis. In addition, by using knockdown and overexpression strategies, we confirmed that SRPK1 can facilitate proliferation, migration, and invasion of CESC cells.

### *mRNA expression and clinical relevance of* SRPK1 *in TCGA database*

**Figure 1.**
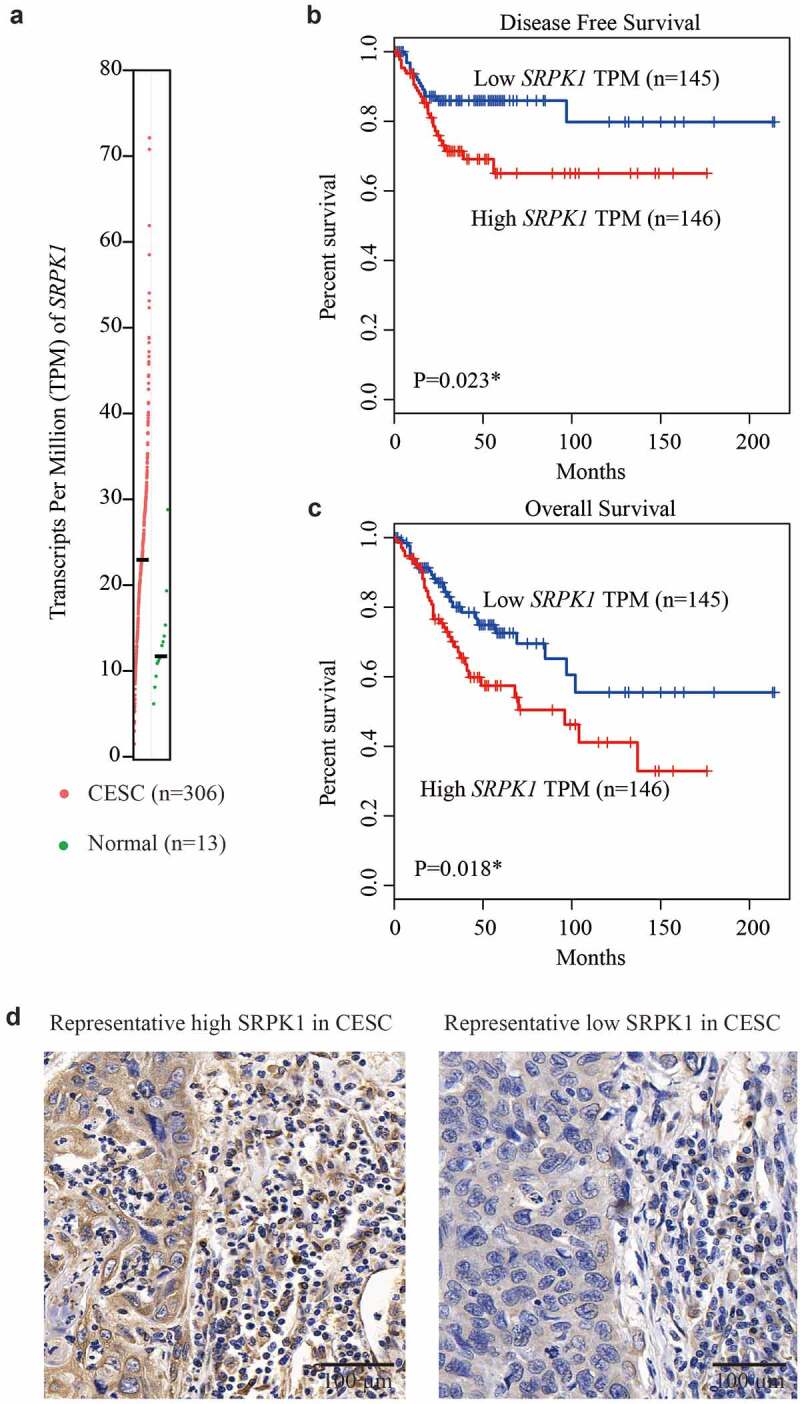
mRNA and protein expression of SRPK1 in CESC samples.

We first extracted the mRNA expression information of *SRPK1* from TCGA database. As reflected by the transcripts per million (TPM), CESC tissues possess a significantly higher *SRPK1*-mRNA level than that of normal cervix tissues (Figure 1a, p < 0.001). In addition, Kaplan–Meier survival curves revealed that patients with higher *SRPK1*-mRNA exhibited worse DFS (P = 0.023, Figure 1b) as well as overall survival (P = 0.018, Figure 1c).

(a) The mRNA level of *SRPK1* was exhibited as transcripts per million (TPM), which was retrieved from TCGA database. Each red dot represents one sample data of CESC (n = 306), each green dot represent one sample data from normal cervix (n = 13). (b) Patients in TCGA database was divided to high-*SRPK1* group (n = 146) and low-*SRPK1* group (n = 145). Kaplan–Meier method was used to generate the disease-free survival curve according to the mRNA level of *SRPK1*. (c) Similarly, the overall survival curves were plotted and tested via log-rank test, which revealed that higher *SRPK1* TPM level indicates poorer overall survival (P = 0.018). (d) Representative IHC images showing the high SRPK1 (left panel) and low SRPK1 (right panel) staining in CESC tissues. Magnification: 400X.

### Patients’ characteristics and protein expression of SRPK1 in enrolled CESC cohort

The distinct expression and clinical relevance of *SRPK1*-mRNA in TCGA database engaged us to further explore its protein expression profiles in our retrospective cohort (n = 122), which contains 74 FIGO stage I cases and 48 FIGO stage II cases that underwent surgical resection in our hospital (Table 1). Among the 122 cases, up to 105 patients were diagnosed with positive HPV (human papillomavirus) infection. There were 78 cases showed smaller horizontal diffusion diameter as less than 4.0 cm, while the other 44 cases with larger tumor size. As for the stromal invasion depth, 63 cases showed less than 2/3 stromal invasion, and the other 59 with ≥ 2/3 stromal invasion depth. The vagina invasion was found to be positive in 28 cases and negative in 94 cases. Similarly, 26 cases showed positive parametrial invasion and 96 cases negative. There were 40 cases identified with positive lymphovascular invasion and 44 cases with positive lymph nodes.

**Table 1.** Correlation between SRPK1 protein expression and clinicopathologic characteristics of CSCC patients

Variables	Cases (n = 122)	SRPK1 protein level		P
		Low (n = 54)	High (n = 68)	
Age (year)				
< 47	53	24	29	0.856
≥ 47	69	30	39	
HPV infection				
Negative	17	6	11	0.600
Positive	105	48	57	
Horizontal diffusion diameter				
< 4.0 cm	78	42	36	0.005*
≥ 4.0 cm	44	12	32	
Stromal invasion depth				
< 2/3	63	26	37	0.585
≥ 2/3	59	28	31	
Vagina invasion				
Negative	94	42	52	0.999
Positive	28	12	16	
Parametrial invasion				
Negative	96	48	48	0.015*
Positive	26	6	20	
Lymphovascular invasion				
Negative	82	41	41	0.082
Positive	40	13	27	
Lymph node metastasis				
Negative	78	42	36	0.005*
Positive	44	12	32	
FIGO stage				
Stage I	74	47	27	<0.001*
Stage II	48	7	41	

P value was analyzed by Fisher Exact test. * indicates P < 0.05 with statistical significance.

The average diagnostic age of our cohort was 47.2 years old, therefore we chose 47 years old as the cutoff for age. As for the horizontal diffusion tumor size, we chose 4.0 cm as the cutoff because it is a critical number for cervical cancer FIGO staging

IHC data showed a predominant cytoplasm localization of SRPK1 in CESC tissues (Figure 1d). Consistent with its mRNA level, SRPK1 protein expression exhibited high diversity in different CESC samples. Therefore, we divided patients into low-SRPK1 group (n = 54) and high-SRPK1 group (n = 68). Chi-square tests revealed that patients with higher SRPK1 level were more prevalent with larger horizontal diffusion diameter (P = 0.005). Moreover, the percentages of parametrial invasion and lymph node metastasis were significantly higher in high-SRPK1 group (P = 0.015 and P = 0.005, respectively). Of note, up to 85.4% (41/48) stage II patients were grouped into high-SRPK1 group, while only 36.5% (27/74) stage I cases showed high-SRPK1 expression, indicating that SRPK1 was positively correlated with CESC tumor progression (P < 0.001, Table 1)

### Prognostic significance of SRPK1 in CESC cohort

**Figure 2.**
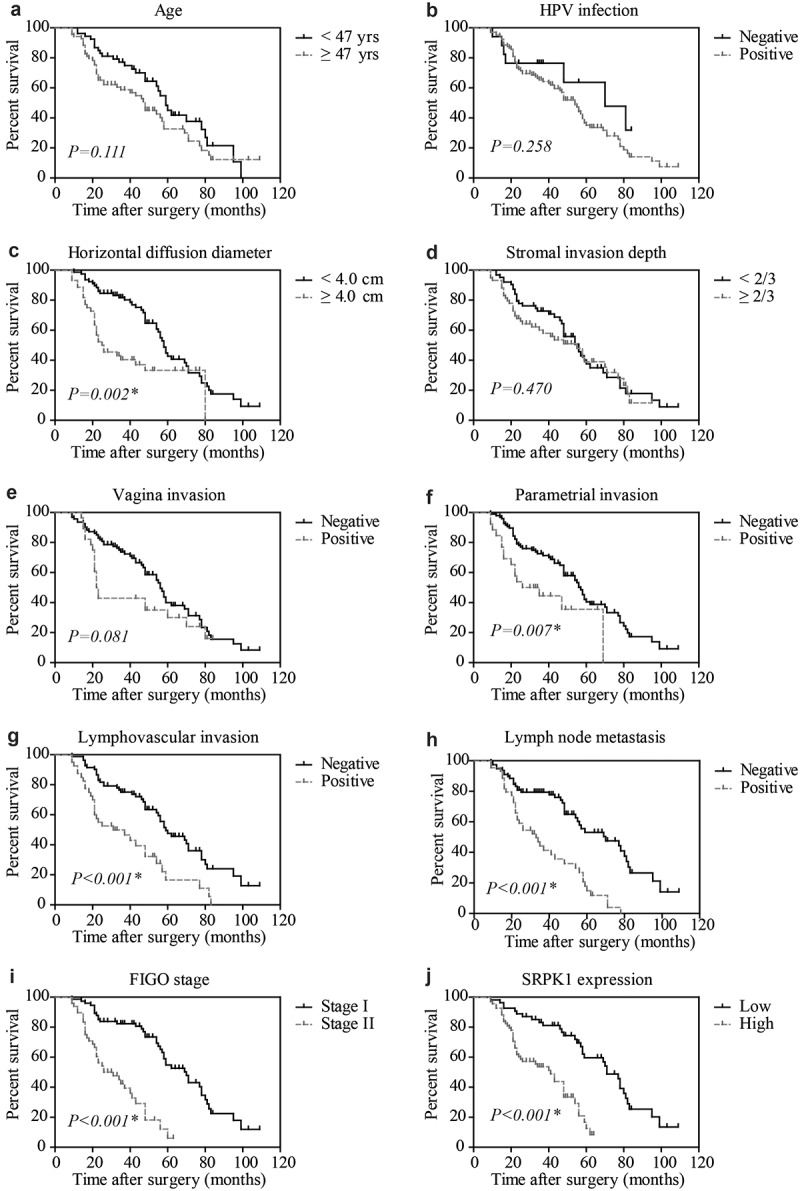
Disease-free survival analyses.

We next assessed the significance of different clinicopathological characteristics on predicting patients’ outcomes according to the DFS curves (Figure 2a–j) and OS curves (Figure S1). Accordingly, high SRPK1 protein expression was remarkably correlated with unfavorable DFS (P < 0.001, Table 2). The 5-year DFS rate was 59.6% in low-SRPK1 group, while decreased to 12.6% in high-SRPK1 group. Consistently, the median DFS time of low-SRPK1 group was 71.0 ± 3.9 months, while decreased to 41.0 ± 5.5 months in high-SRPK1 group (Figure 2j).

Kaplan–Meier method was utilized to evaluate the prognostic significances of all variables, including age (a), HPV infection (b), horizontal diffusion diameter (c), stromal invasion depth (d), vagina invasion (e), parametrial invasion (f), lymphovascular invasion (g), lymph node metastasis (h), FIGO stage (i), and SRPK1 expression (j). Data was analyzed by Kaplan–Meier method, and survival curves were compared by long-rank test. * P < 0.05.

**Table 2.** Disease-free survival (DFS) information of enrolled CSCC patients

Variables	Cases(n = 122)	Univariate analysis			Multivariate analysis	
		5-year DFS rate (%)	Median DFS time (months)	P	HR (95% CI)	P
Age (year)						
< 47	53	45.1%	59.0 ± 3.6	0.111		
≥ 47	69	32.7%	48.0 ± 6.4			
HPV infection						
Negative	17	63.7%	70.0 ± 18.2	0.258		
Positive	105	35.1%	54.0 ± 3.8			
Horizontal diffusion diameter						
< 4.0 cm	78	42.7%	58.0 ± 1.9	0.002*	Reference	0.014*
≥ 4.0 cm	44	33.3%	25.0 ± 6.0		1.96 (1.15–3.35)	
Stromal invasion depth						
< 2/3	63	37.6%	56.0 ± 5.1	0.470		
≥ 2/3	59	39.0%	55.0 ± 8.8			
Vagina invasion						
Negative	94	39.9%	56.0 ± 4.1	0.081		
Positive	28	30.1%	22.0 ± 1.1			
Parametrial invasion						
Negative	96	40.4%	57.0 ± 4.0	0.007*	Reference	0.028*
Positive	26	35.6%	26.0 ± 9.6		1.98 (1.08–3.64)	
Lymphovascular invasion						
Negative	82	47.6%	59.0 ± 6.8	<0.001*	Reference	<0.001*
Positive	40	16.6%	31.0 ± 11.2		2.69 (1.67–4.35)	
Lymph node metastasis						
Negative	78	53.1%	70.0 ± 12.8	<0.001*	Reference	0.002*
Positive	44	14.9%	33.0 ± 5.6		2.35 (1.37–4.02)	
FIGO stage						
Stage I	74	54.8%	70.0 ± 5.7	<0.001*	Reference	0.036*
Stage II	48	6.1%	26.0 ± 6.2		1.90 (1.04–3.47)	
SRPK1 expression						
Low	54	59.6%	71.0 ± 3.9	<0.001*	Reference	0.047*
High	68	12.6%	41.0 ± 5.5		1.88 (1.01–3.50)	

P value was analyzed by log-rank test. * indicates P < 0.05 with statistical significance.

Meanwhile, patients with larger tumor horizontal diameter exhibited worse DFS (median DFS time 25.0 ± 6.0 months vs 58.0 ± 1.9 months, P = 0.002, Figure 2c). The 5-year DFS rate of patients with positive parametrial invasion was 35.6%, while reached to 40.4% of those with negative parametrial invasion (P = 0.007, Figure 2f). Similarly, lymphovascular invasion (Figure 2g) and lymph node metastasis (Figure 2h) were also identified as unfavorable prognostic biomarkers (P < 0.001, Table 2). As expected, patients with FIGO stage II exhibited significantly poorer survival compared to those with FIGO stage I (median DFS time 26.0 ± 6.2 months vs 70.0 ± 5.7 months, P < 0.001, Figure 2i). Consistently, all the above significant prognostic factors for DFS also showed significance in OS curves (Figure S1). In contrast, patients’ age (Figure 2a), HPV infection (Figure 2b), stromal invasion depth (Figure 2d), and vagina invasion (Figure 2e) showed no statistically significant effect on patients prognosis.

A Cox proportional hazards regression model was further selected to validate the independent effect of each significant predictor of DFS (Table 2). Among them, SRPK1 was confirmed as a novel independent prognostic factor with hazard ratio as 1.878 (95% CI 1.008–3.501, P = 0.047). Besides higher SRPK1, larger horizontal diffusion diameter (HR = 1.961, 95% CI = 1.149–3.349, P = 0.014), positive parametrial invasion (HR = 1.979, 95% CI = 1.075–3.641, P = 0.028), positive lymphovascular invasion (HR = 2.963, 95% CI = 1.666–4.354, P < 0.001), positive lymph node metastasis (HR = 2.349, 95% CI = 1.373–4.019, P = 0.002), and advanced FIGO stage (HR = 1.899, 95% CI = 1.041–3.465, P = 0.036) all showed independent prognostic significance. Of note, higher SRPK1 can also independently predict a worse overall survival of CESC patients (HR = 1.868, 95% CI = 1.025–3.667, P = 0.014; Table 3).

**Table 3.** Overall survival (OS) information of enrolled CSCC patients

Variables	Cases(n = 122)	Univariate analysis			Multivariate analysis	
		5-year OS rate (%)	Median OS time (months)	P	HR (95% CI)	P
Age (year)						
< 47	53	58.9%	83.0 ± 17.1	0.204		
≥ 47	69	51.8%	62.0 ± 5.5			
HPV infection						
Negative	17	70.6%	99.0 ± 0	0.369		
Positive	105	52.9%	66.0 ± 4.8			
Horizontal diffusion diameter						
< 4.0 cm	78	64.1%	72.0 ± 5.1	0.008*	Reference	0.053
≥ 4.0 cm	44	38.6%	36.0 ± 11.2		1.77 (0.99–3.16)	
Stromal invasion depth						
< 2/3	63	60.4%	69.0 ± 10.5	0.175		
≥ 2/3	59	48.8%	59.0 ± 9.5			
Vagina invasion						
Negative	94	59.2%	69.0 ± 5.4	0.060		
Positive	28	39.8%	30.0 ± 20.1			
Parametrial invasion						
Negative	96	59.6%	72.0 ± 7.3	0.006*	Reference	0.023*
Positive	26	34.9%	47.0 ± 13.4		2.10 (1.11–3.96)	
Lymphovascular invasion						
Negative	82	64.3%	80.0 ± 7.4	<0.001*	Reference	<0.001*
Positive	40	34.6%	46.0 ± 11.9		2.90 (1.71–4.92)	
Lymph node metastasis						
Negative	78	65.0%	83.0 ± 9.7	<0.001*	Reference	0.005*
Positive	44	35.9%	43.0 ± 5.9		2.30 (1.30–4.10)	
FIGO stage						
Stage I	74	72.3%	80.0 ± 7.1	<0.001*	Reference	0.044*
Stage II	48	22.0%	41.0 ± 5.5		1.26 (1.02–2.49)	
SRPK1 expression						
Low	54	69.7%	80.0 ± 7.4	0.001*	Reference	0.042*
High	68	41.3%	54.0 ± 8.4		1.87 (1.03–3.67)	

P value was analyzed by log-rank test. * indicates P < 0.05 with statistical significance.

### Overexpressing SRPK1 promotes CESC proliferation, migration, and invasion

**Figure 3.**
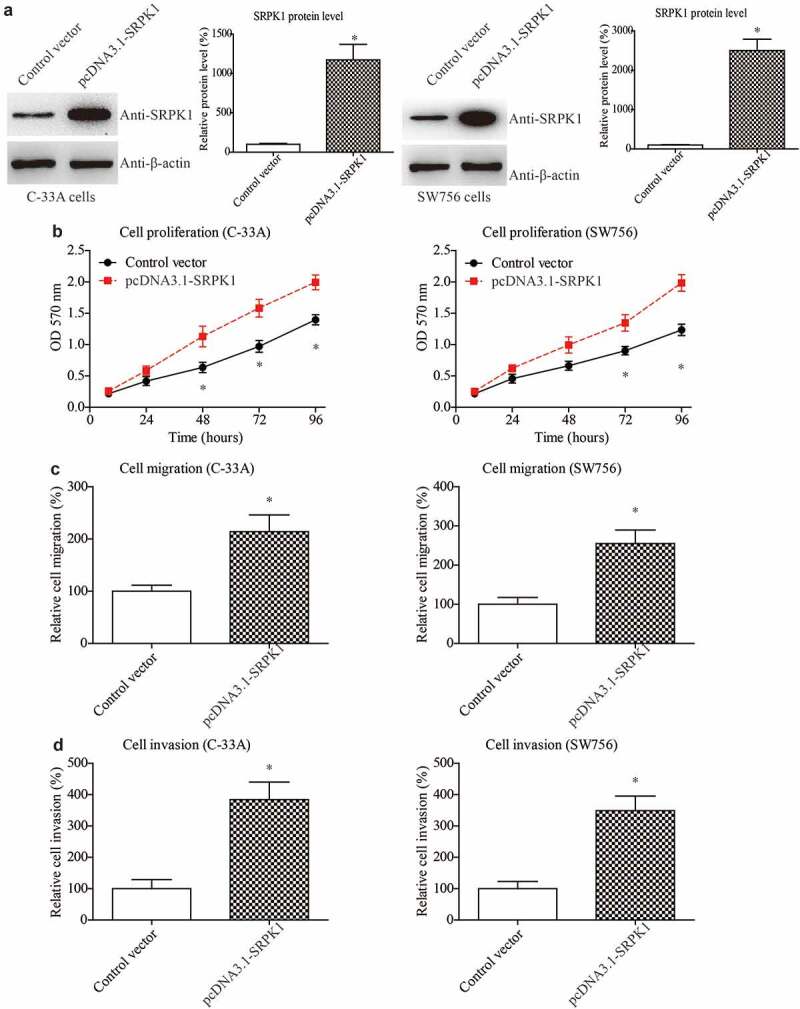
Overexpressing SRPK1 promotes CESC proliferation, migration, and invasion.

Considering that the staging system is closely correlated to the tumor size, invasion, and metastasis, these tumor progression processes are therefore the major factors affecting patients’ prognosis. Therefore, we conducted cellular experiments to validate the role of SRPK1 in CESC cells. We firstly overexpressed SRPK1 in two CESC cell lines, C-33A and SW756. Western blot was used to test the transfection efficiencies, which demonstrated successful overexpression in both cell lines (Figure 3a). Next, we evaluated cell proliferative capacity by using MTT strategy. As a result, SRPK1-overexpression remarkably enhanced the cell viability according to the growth curve (Figure 3b). Meanwhile, migration and invasion abilities were assessed by Transwell method. Accordingly, SRPK1-transfection led to a significant increase on the migrated cell numbers of both C-33A and SW756 cells (Figure 3c). Consistently, the number of invaded cells were also significantly higher in SRPK1-transfected groups compared to the control groups in both cell lines (Figure 3d).

(a) The transfection efficiencies were tested via Western blot in both C-33A and SW756 cells. (b) MTT assays were used to plot the cell proliferation curves, showing the significant effect of SRPK1 on facilitating CESC cell proliferation. (c) Transwell experiments demonstrated that SRPK1-transfection can enhance CESC migration capacity. (d) Matrigel-Transwell data revealed the role of SRPK1 on promoting CESC invasion.Data were obtained from three independent repeats and presented as mean + SD. * P < 0.05 by Student’s t-test.

### SRPK1 interference results in attenuated CESC viability

**Figure 4.**
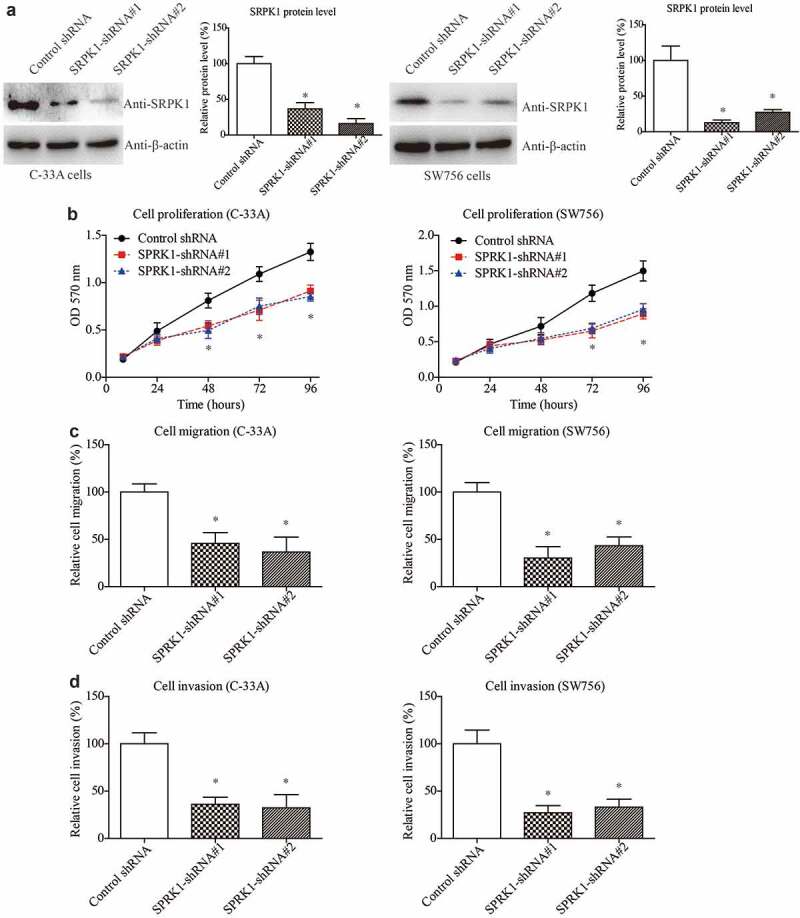
SRPK1 interference results in attenuated CESC viability.

To better illustrate the potential of SRPK1 as a therapeutic target, we next silenced SRPK1 using shRNAs in CESC cells (Figure 4a). As expected, SRPK1-knockdown resulted in an opposite effect on the cell proliferation curves in both C-33A and SW756 cell lines (Figure 4b). In addition, silencing SRPK1 can significantly inhibit the migration and invasion processes of CESC cells (Figure 4c,d). Taken together, our cellular experiments demonstrated the novel effects of SRPK1 on positively regulating CESC cell proliferation, migration, and invasion. The cellular findings are consistent with its clinical relevance with tumor diameter and parametrial invasion (Table 1).

(a) Knockdown efficiencies of shRNAs were tested via Western blot in both C-33A and SW756 cells. (b) MTT assays showed significant effects of SRPK1-shRNAs on inhibiting CESC cell proliferation. (c) Transwell data demonstrated that silencing SRPK1 suppressed CESC migration. (d) Matrigel-Transwell results validated the invasion-suppressing role of SRPK1 interference. Data were obtained from three independent repeats and presented as mean + SD. * P < 0.05.

## Discussions

Here in the current study, we initially investigate the expression and tumor-related function of SRPK1 in cervical cancer. According to our data, SRPK1 can promote the proliferation, migration, and invasion of CESC, thus promoting tumor progression. Our data are consistent with its reported role in hepatocellular carcinoma (HCC). Overexpression of wild-type SRPK1 promoted HCC cell proliferation while forced expression of its kinase-dead mutant or silencing SRPK1 resulted in attenuated tumor growth both in vitro and in vivo [22]. However, SRPK1 seems to play more complicated functions in various malignancies. For example, besides enhancing tumor cell proliferation, elevated SRPK1 can also attenuate apoptosis of breast cancer cells and esophageal squamous cell carcinomas [30,31]. SRPK1 overexpression can also induce stem cell-like phenotype in non-small-cell lung carcinoma [32]. Another function of SRPK1 is tumor-related angiogenesis [33]. As reported, inhibition or knockdown of SRPK1 can remarkably prevent in vitro and in vivo angiogenesis and associated with tumor growth of Wilms’ tumor [34] and prostate cancer [35], highlighting its potential as a novel drug target.

Additionally, SRPK1 also participates in chemotherapy resistance. On one hand, higher SRPK1 increased oxaliplatin-resistant of colon cancer cells [36,37], and knockdown of SRPK1 enhanced ovarian cancer sensitivity to cisplatin [38]. Similarly, inhibition of SRPK1 by SRPIN340, its specific inhibitor, resulted in antileukemia effects [39]. In contrast, the function of SRPK1 seems different in testicular germ cell tumors. According to the data by Schenk et al., low SRPK1 expression is significantly correlated with resistance to platinum-containing chemotherapy in testicular germ cell tumors, thus leading to worse prognosis [40]. Similarly, decreased SRPK1 may also lead to the cisplatin-resistance of retinoblastoma [41], indicating the complicated crosstalk between SRPK1 and chemotherapy resistance. These contradictory findings may be at least partially explained by the findings by Wang et al., which report that SRPK1 can function as both an oncogene and a tumor suppressor by modulating the PH domain leucine-rich repeat protein phosphatases (PHLPP)-mediated dephosphorylation of protein kinase B [42], its highly likely that the PHLPP-mediated downstream pathways may also participated in the chemotherapy-resistance. Nevertheless, more and more studies are now focusing on developing SRPK1 inhibitors to test the tumor-suppressing functions [43,44]. Besides small-molecule inhibitors, chimeric antibody targeting SRPK1 also inhibits non-small cell lung cancer progression on multiple aspects [45]. Our findings on the tumor-suppressing role by SRPK1-knockdown may provide novel insights for CESC research and guidance for individual therapy. Therefore, our data not only identified a novel prognostic factor for CESC, but also provided a potential therapeutic target. Focusing on SRPK1 specific inhibitors deserve further investigation on cancer treatment.

One limitation of this study is the lack of investigating signaling mechanisms. Further studies will be essential to further explore the multifaced mechanism of SRPK1 in different malignancies. As the major substrates of SRPK1, the expression of SRSFs (serine/arginine-rich splicing factors) were also altered in malignances in accordance with SRPK1 [46], indicating the SRPK1 may modulate tumor progression by modulating the alternative splicing process. Interestingly, a recent study by Sarah et al. demonstrated that human papillomavirus type 16 infection can stimulate the host SRPK1-SRSF axis via the viral E2 protein in keratinocytes [47]. However, our clinical cohort did not find any significant correlation between SRPK1 expression with HPV infection. This may be partially due to the limited case numbers of HPV-negative patients. Nevertheless, we proved its independent prognostic role in the recurrence and survival of CESC. Another limitation is that all the cases for IHC analyses were obtained from our medical center, which may result in regional bias. We tried to make our conclusion more convincible by analyzing mRNA level and role of SRPK1 from TCGA database. However, future studies will be necessary to validate our findings in multiple centers.

## Conclusions

We identified and validated the high SRPK1 expression as an independent risk factor for prognosis of CESC patients. Moreover, based on the cellular experiments, SRPK1 may serve as a potential therapeutic target in CESC.

## Supplementary Material

Supplemental Material

## Highlights

- SRPK1 is aberrantly up-regulated in CESC tissues than that in normal cervix tissues.
- Higher SRPK1 can help predict worse prognosis of CESC patients.
- SRPK1 can positively regulate CESC cell proliferation and invasion.

## Abbreviations

CESC (cervical squamous cell carcinoma), FIGO (The International Federation of Gynecology and Obstetrics), GEPIA (Gene expression profiling interaction analysis), HCC (hepatocellular carcinoma), HPV (Human papillomavirus), IHC (immunohistochemistry), MTT (3-(4,5-Dimethylthiazol-2-yl), OS (overall survival), DFS (disease-free survival), PHLPP (PH domain leucine-rich repeat protein phosphatases), SD (standard deviation), SRPK1 (serine/arginine-rich protein-specific kinase 1), SRSF (serine/arginine-rich splicing factor), TCGA (The Cancer Genome Atlas), TPM (transcripts per million).
