# Supplementary material for: Increased expression of SRPK1 (serine/arginine-rich protein-specific kinase 1) is associated with progression and unfavorable prognosis in cervical squamous cell carcinoma
Source: Bioengineered. 2022 Feb 22;13(3):6100–12. doi: 10.1080/21655979.2022.2034705 (PMC8973769; doi:10.1080/21655979.2022.2034705)
Supplement: Supplemental Material [file KBIE_A_2034705_SM1618.docx]

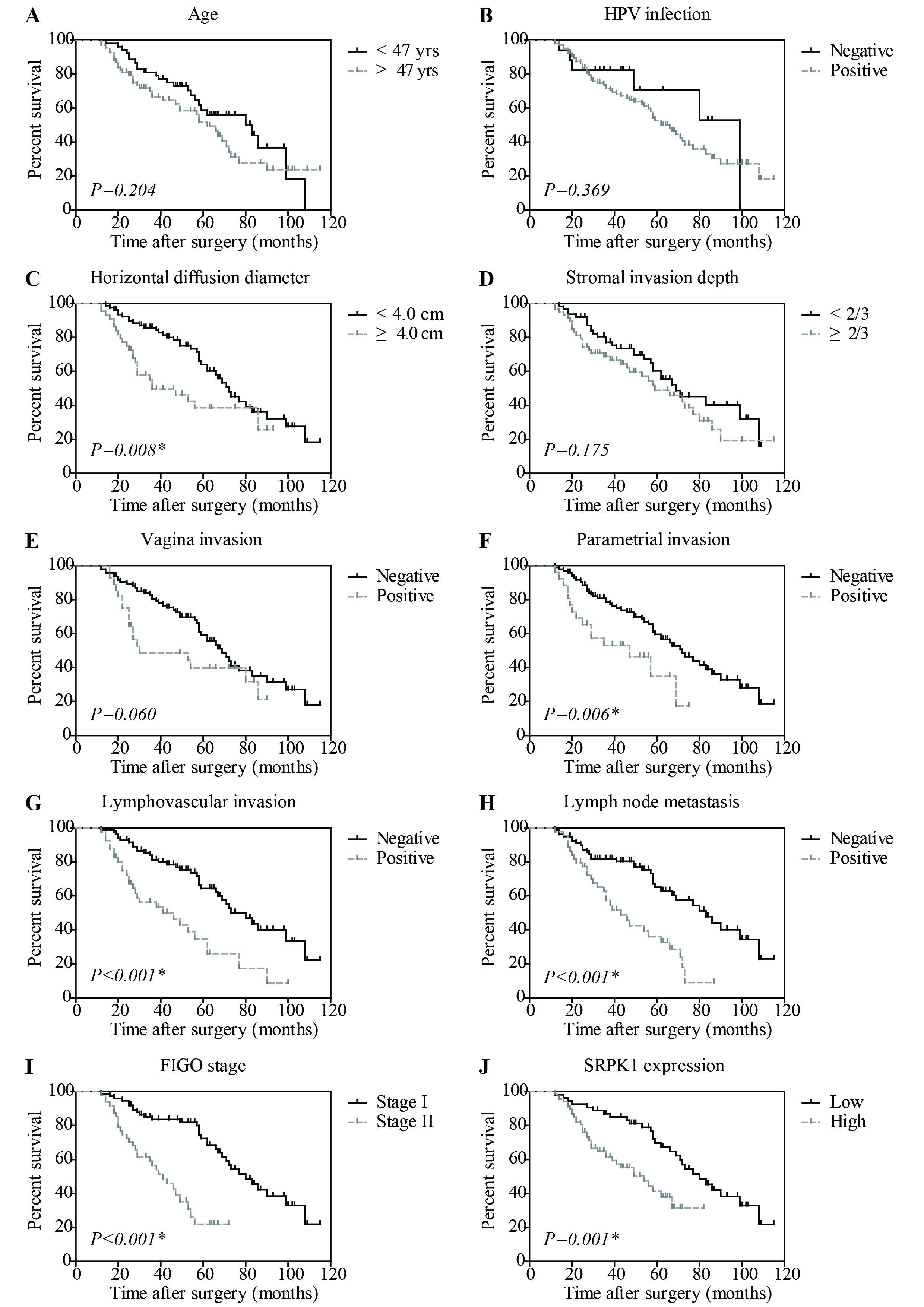


**Figure S1. Overall survival analyses.**

Kaplan-Meier method was utilized to evaluate the prognostic significances of all variables, including age (A), HPV infection (B), horizontal diffusion diameter (C), stromal invasion depth (D), vagina invasion (E), parametrial invasion (F), lymphovascular invasion (G), lymph node metastasis (H), FIGO stage (I), and SRPK1 expression (J). Data was analyzed by Kaplan-Meier method, and survival curves were compared by long-rank test. * P<0.05.
